# 27.1 TRANSLATIONAL EVIDENCE OF DOPAMINE-RELATED ALTERATIONS OF BASAL GANGLIA AND THALAMO-CORTICAL NEUROCIRCUITRY IN SCHIZOPHRENIA: A FULL CLINIC-TO-BENCH-TO-CLINIC BACK-TRANSLATION

**DOI:** 10.1093/schbul/sby014.111

**Published:** 2018-04-01

**Authors:** Jared Van Snellenberg, Guillermo Horga, Roberto Gil, Christoph Kellendonk, Anissa Abi-Dargham

**Affiliations:** 1 Stony Brook University Medical School; 2 Columbia University/New York State Psychiatric Institute

## Abstract

**Background:**

Recent work with a dopamine 2 receptor (D2R) over-expressing (D2R-OE) mouse has suggested that this receptor over-expression leads to a highly plastic increase in bridging collaterals from the associative striatum (AST) to the external segment of the globus pallidus (GPe). Because of the densely interconnected nature of basal ganglia-thalamo-cortical signaling circuitry, we hypothesized and demonstrated in a recent publication that the resting state functional connectivity (RSFC) of AST to multiple cortical and thalamic subregions is broadly disrupted in unmedicated patients with schizophrenia. In this talk, I will present novel simultaneous multi-slice (aka “multiband”) fMRI data that provides the spatial resolution necessary to image smaller basal ganglia substructures (such as the GPe/GPi), and show that unmedicated patients with schizophrenia exhibit specifically disrupted AST-GPe connectivity, as predicted directly from the D2R-OE mouse model findings.

In addition, recent work with a 22q11 deletion mouse, which models a similar syndrome in humans that is strongly associated with schizophrenia, has shown that these mice exhibit a D2R-mediated reduction in the strength of excitatory post-synaptic potentials in primary auditory cortex in response to stimulation of the medial geniculate nucleus (MGN) of the thalamus. Consistent with this finding, I will present multiband fMRI data that employs both RSFC and an audio-visual localizer task to demonstrate a specific reduction in RSFC between the MGN and primary auditory cortex, consistent with these findings in the 22q11 mouse.

**Methods:**

Partially-overlapping samples of 19 and 14 unmedicated patients with schizophrenia and 15 and 16 matched healthy participants participated in two sets of studies. For both studies, multiband fMRI images were acquired on a GE MR 750 system at the New York State Psychiatric Institute, with a multiband acceleration factor of 6, 2 mm isotropic voxel resolution, and 850 ms TR. Thirty minutes of RSFC data was collected in each participant, and participants in the auditory study also completed a 15 minute audio-visual localizer task that employed sparse temporal sampling with either auditory (9 seconds of a randomized and rapidly-varying musical stimulus) or visual (7.5 Hz alternating checkerboard) stimulation between each acquisition cluster. Basal ganglia subregions were identified via manual drawings conducted by an experienced rater, and the MGN and LGN were identified using the audio-visual localizer task.

**Results:**

Unmedicated patients with schizophrenia showed a significant reduction in RSFC strength between the dorsal caudate and GPe (Cohen’s d = 0.87, P = 0.017), but no other striatal or pallidal subregion pairs, consistent with a specific alteration in anatomical projections between these two regions. In addition, patients with schizophrenia showed a reduction in RSFC between the MGN and primary auditory cortex, as well as between the LGN and primary visual cortex (P < 0.05, alphasim corrected for whole-brain analysis).

**Discussion:**

These findings provide initial support for the existence of D2R-mediated alterations in functional neuroanatomy, first observed in animal models of schizophrenia, in a clinical sample of unmedicated patients. In addition to providing early evidence for potential mechanisms of psychotic phenomena, this work suggests that the use of non-invasive multiband RSFC is a promising approach to translating basic neuroscience findings in animal models back into a clinical setting. Altered circuitry was shown in the D2OE mice to underlie motivational deficits, and we propose that they may have a similar functional impact in patients.

